# Multiscale Sample Entropy-Based Feature Extraction with Gaussian Mixture Model for Detection and Classification of Blue Whale Vocalization

**DOI:** 10.3390/e27040355

**Published:** 2025-03-28

**Authors:** Oluwaseyi Paul Babalola, Olayinka Olaolu Ogundile, Vipin Balyan

**Affiliations:** 1 French-South African Institute of Technology, Department of Electrical, Electronic, and Computer Engineering, Cape Peninsula University of Technology, Bellville, Cape Town 7535, South Africa; 2 Department of Industrial Technical Education, Tai Solarin University of Education, Ijebu-Ode 2118, Ogun State, Nigeria

**Keywords:** blue whale, classification, detection, DMD, GMM, MSE, PCA, WF, vocalization

## Abstract

A multiscale sample entropy (MSE) algorithm is presented as a time domain feature extraction method to study the vocal behavior of blue whales through continuous acoustic monitoring. Additionally, MSE is applied to the Gaussian mixture model (GMM) for blue whale call detection and classification. The performance of the proposed MSE-GMM algorithm is experimentally assessed and benchmarked against traditional methods, including principal component analysis (PCA), wavelet-based feature (WF) extraction, and dynamic mode decomposition (DMD), all combined with the GMM. This study utilizes recorded data from the Antarctic open source library. To improve the accuracy of classification models, a GMM-based feature selection method is proposed, which evaluates both positively and negatively correlated features while considering inter-feature correlations. The proposed method demonstrates enhanced performance over conventional PCA-GMM, DMD-GMM, and WF-GMM methods, achieving higher accuracy and lower error rates when classifying the non-stationary and complex vocalizations of blue whales.

## 1. Introduction

Marine mammals require conservation and protection from threats brought on by a variety of factors, including rising ocean temperatures from climate change, disturbances and accidents, pollution, and other damaging human activities. According to [1], blue and fin whale species are specifically classified as critical, with Antarctic blue whales being particularly endangered. Thus, there is a need to develop effective monitoring schemes to examine the existence and numbers of species, as well as observe the potential implications of human activity and take measures for ecological preservation.

Recently, passive acoustic monitoring (PAM) [1] has emerged as a successful method for observing whales without interfering with the animal’s behavior. The advancement of autonomous underwater sensors, which are typically incorporated into monitoring equipment for predicting long-term patterns and seasonal variation and evaluating the consequences of environmental degradation and human activity, has expanded the potential of PAM [1]. Traditionally, whale vocalizations from recordings were manually identified and reported by either observing the whale’s signal on a spectogram or having marine ecology experts listen to the sounds. However, the manual detection approach is susceptible to human error and impractical, given the volume of modern datasets [2]. Thus, different automatic detection approaches have emerged for detection of whale vocalizations through sound.

Whale vocalizations are considered non-stationary due to their significant variability and complexity over time [3,4]. The variability in whale sounds can be attributed to several factors, such as the purpose of the sound, the individual whale producing the sound, and environmental conditions. These vocalizations can vary in frequency, amplitude, and duration, making it challenging to apply stationary methods to analyze them effectively [5].

One approach to quantifying the complexity and variability of non-stationary signals such as whale sounds is through the use of entropy measures. Conventional sample entropy (SE) [6,7,8], however, may not be appropriate for analyzing whale sounds due to their non-stationary nature. Sample entropy serves as a valuable metric for assessing the degree of regularity and structural complexity within time series data. It estimates the probability of pattern repetition within a given tolerance, making it sensitive to changes in the underlying dynamics of the time series.

Given the non-stationary nature of whale sounds, nonlinear methods of complexity analysis may be better suited for capturing their variability and complexity compared with traditional linear methods. Nonlinear methods, such as permutation entropy (PermEnt) [9,10,11], are advantageous in this context because they can effectively capture the complex, nonlinear patterns and interactions present in non-stationary signals. In contrast, traditional linear methods may not fully capture these complex dynamics, potentially leading to an incomplete characterization of the underlying patterns in whale sounds. In [10], PermEnt and statistical complexity were incorporated for the automatic detection of fish vocalizations. This method of detection showed a good degree of detection accuracy that was not severely affected by the presence of common background noises from human and natural sources in the recordings. Furthermore, Shishidhar et al. [11] expanded on the findings in [10] for the detection of dolphin whistles and clicks using PermEnt and SE, respectively. However, the approach utilized a fixed-threshold approach for binary classification of the whale calls.

To better understand the characteristics of blue whale vocalization, including patterns of communication, vocal repertoire, acoustic properties, and behavioral adjustments in reaction to environmental influences, meaningful attributes (features) must be extracted from the acoustic data through the application of feature extraction methods. In the literature [12,13], diverse time-domain parameters like the mean values, amplitude, and zero crossing rates have been employed to characterize distinct whale vocalizations. Similarly, features within the frequency domain, like spectral characteristics, cepstral features, and wavelet transforms [14,15,16], have been utilized to depict whale vocal signals.

Furthermore, different methods for feature reduction have been employed to eliminate redundant attributes from the feature space while preserving the pertinent information. These methods include dynamic mode decomposition (DMD) [17,18], principal component analysis (PCA) [19,20], wavelet-based feature (WF) [21], and other eigen-based decomposition techniques [22]. These methods aid in enhancement of the robustness of machine learning tools, encompassing the hidden Markov model (HMM) [18,20], Gaussian mixed model (GMM) [23], support vector machines (SVMs) [24], neural networks (NNs), and their variations [25,26], thus enhancing their capacity to handle and process data effectively.

In this study, a multiscale sample entropy (MSE) algorithm is presented as a time-domain feature extraction method to examine blue whale acoustic signals in continuous acoustic recordings. The MSE algorithm quantifies the intricacy of dynamic signals by evaluating their entropy at various temporal scales. Since its introduction in the field of pysiological study [27], different enhancements and adaptations are suggested in the literature [28] to improve the precision of entropy estimates, investigate alternative coarse-graining techniques, and expand the methodology’s capabilities. However, the MSE is employed to extract useful features from blue whale vocalization.

Furthermore, the MSE-based features are adopted to the GMM to reduce the dimensionality of the extracted features and cluster similar calls and events within the data. The GMM categorizes signals into two groups—whale calls and other sources of sounds—encompassing background noise originating from factors such as rain, sea currents, drifting icebergs, and anthropogenic activities [29]. In addition to classification, the GMM helps eliminate the need for an entropy threshold that is usually manually determined by the user in existing studies. The performance of the proposed MSE feature extraction method is assessed through empirical evaluation, with its performance benchmarked against established PCA, DMD, and WF methods using real-world data from [30]. Key performance metrics, including accuracy and error rate, are employed to quantify the relative effectiveness of these methods.

The remainder of this work is as follows. General descriptions of the whale vocalizations and different call types are presented in Section 2.1. Section 2.3 discusses the preprocessing steps involved in preparing the recorded acoustic signal for further analysis. The existing feature extraction approaches for large and complicated datasets are outlined in Section 2.4. Section 2.6 explores the proposed MSE-based feature extraction method. Section 2.7 describes a technique for feature reduction utilizing the GMM. The methodology for training, testing, and classification using the GMM is detailed in Section 2.8 and Section 2.9. The experimental set-up is given in Section 3, while the simulation results from the experiments on continuous recordings and the discussion are outlined in Section 4. A conclusion summarizing this study’s findings and outlining future work directions is provided in Section 5.

## 2. Methods

### 2.1. Blue Whale Calls

Antarctic blue whales, scientifically classified as *Balaenoptera musculus intermedia*, are noted for their unique vocalizations, which aid in communication and social activities. The calls reveal important information about whale behavior, locations, and trends in populations in the confines of the Southern Ocean [31,32,33]. Remarkably, the vocalizations produced by these whales rank among the loudest generated by any mammal, ranging between 186 and 189 dB re 1 μPa [31]. These calls possess the ability to traverse substantial distances underwater, spanning a range of 200–1700 km [34]. Characteristically, the calls feature predominantly low-frequency components and exhibit a spectrum ranging from simple, repetitive patterns to more intricate and changeable sequences [31]. Notably, these calls can function as distictive acoustic patterns, enabling the distinct identification and long-term tracking of whale movements [2].

Blue whale vocalizations encompass both songs or moans [35] and non-songs [2]. These songs are specific to certain populations, as indicated in [30,31,36,37]. In particular, Antarctic blue whales exhibit a distinctive vocal behavior during their breeding season, characterized by the production of stereotypical Z calls [38,39]. This unique vocalization is integral to male mating displays, as they engage in competitive singing to capture the attention of potential female mates. Z calls consist of three segments with frequency modulation and durations spanning from 18 to 26 s [30,37,40]. The first segment, identified as *Bm-Ant-A* in [30], has a duration of 8–12 s and occurs within a frequency range of 25–27 Hz. The second Z call segment has a characteristic downsweep that ranges from about 27 Hz to 20 Hz and lasts for 2 s. This specific call portion was labeled *BM-Ant-B* in [30], which is used to identify a subset of Z calls having the *Bm-Ant-A* portion alongside a partial or full downsweep. The final portion, which lasts 8–12 s, has frequency modulation ranging from 20 Hz to about 18 Hz [41] and is denoted as *Bm-Ant-Z* in [30].

Conversely, the non-song vocalizations known as D calls are a separate and unique class of signals distinguished by their unpredictability and significant frequency modulation [2,30]. These calls exhibit a wide frequency range, ranging between 22 Hz and 106 Hz, and have extended vocal durations, often lasting from about 1 s to 4 s [30,36,39]. It has been previously posited by various authors that the D-call types are vocalizations emitted by blue whales of both sexes during feeding activities, implying a potential role in foraging communication. However, a recent investigation [42] showed an intriguing aspect of D calls. This study found that these calls are not solely associated with feeding behaviors and are also produced in the context of mating activities among blue whales.

### 2.2. Data Description and Annotations

The blue whale audio recordings from the dataset [30] were utilized in this study. These recordings were captured from seven survey sites in the Antarctic region and serve as the primary dataset for evaluating and analyzing the proposed multiscale sample entropy-based GMM classification algorithm. The underwater sound recordings from the Antarctic region have been carefully examined and annotated, forming a comprehensive library of reference data. This annotated library is accessible through the IWC-SORP/SOOS Annotated Library [43]. This resource not only contains a large collection of audio recordings but also offers useful annotations and contextual information that will greatly enhance the precision and reliability of the analysis in this study.

Balleny Island, one of the seven sites where blue whale recordings were acquired by Miller et al. [30], is a focal point in this study. Positioned at the coordinates 65°21.34′ S, 167°54.69′ E, the island is located within the Antarctic region in the Southern Ocean. The analysis centers on a comprehensive dataset of blue whale recordings from the island, spanning an entire year from January 2015 to January 2016. This dataset comprises 204 annotated recordings, each carefully examined to gain insights into blue whale vocalizations.

Raven Pro 1.6.5 software [44] was utilized for acoustic analysis, facilitating manual identification and annotation of the calls. This meticulous examination ensured accurate analysis of the recordings. The spectrogram settings in Raven Pro were optimized by selecting a Hann window with a length of 1024 samples. This window function balances the frequency resolution and leakage, providing a clear representation of the frequency content in the recordings. The time grid parameters were set to achieve a 50% overlap between consecutive windows, with a hop size of 514 samples. This overlap captured the temporal dynamics of the vocalizations while maintaining a reasonable computational load. Furthermore, the spectrogram was configured with a 3-dB filter bandwidth of 1.40 Hz, reducing noise and emphasizing the signal’s frequency components. A discrete Fourier transform (DFT) size of 2048 samples was employed to achieve a satisfactory trade-off between the frequency resolution and computational efficiency. Finally, the grid spacing was set to 0.488 Hz, allowing for a detailed examination of the frequency content in the recordings.

**Figure 1:**
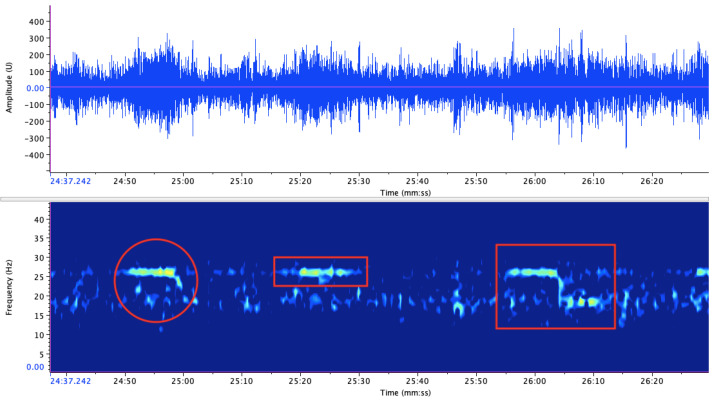
Waveform and spectrogram views of blue whale vocalizations: Bm-Ant-A (rectangle), Bm-Ant-B (circle), and Bm-Ant-Z (square).

Figure 1 provides a visualization of the vocalization samples from Balleny Island attributed to *Bm-Ant-A* (rectangle), *Bm-Ant-B* (circle), and *Bm-Ant-Z* (square). These vocalizations were thoroughly documented and discussed in [30,43].

### 2.3. Data Preprocessing

We start with the recorded acoustic signal as a continuous time series

$$x\left(t\right)=\left\{x_{t};t=1,2,\ldots ,N\right\},$$

where *N* denotes the sample size of the dataset.

**Figure 2:**
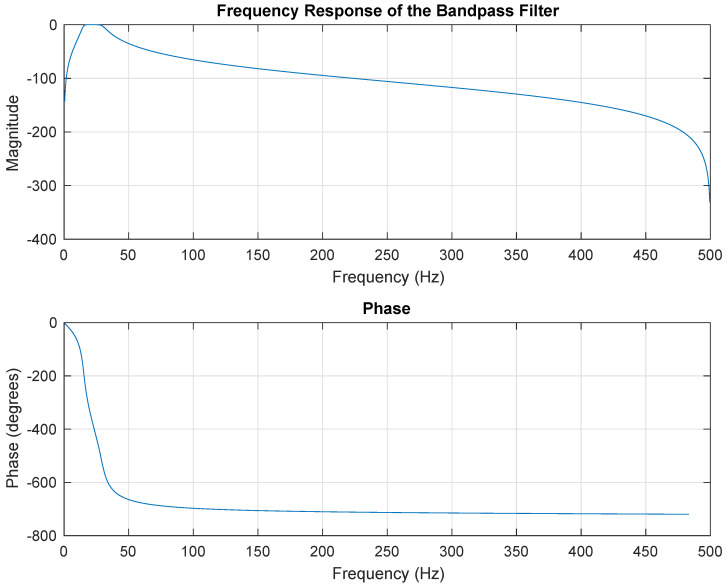
Frequency response of the bandpass filter: (**lower**) cutoff frequency of 15 Hz and (**upper**) cutoff frequency of 30 Hz.

The initial step involves the use of a bandpass filter [45]. This specialized filter is intended to selectively eliminate any undesired low- and high-frequency noise in the recorded data. The filter design is aimed to match the distinctive characteristics of the signal and the expected frequency range associated with blue whale calls. According to the relevant literature, these calls can span a frequency range from 15 Hz to 30 Hz. Hence, the bandpass filter removes noise outside of the 15–30 Hz band. The filtered signal is expressed as [45]

(1) $$y\left(t\right)=\left(\frac{1}{2\pi }\right)\int H\left(f\right)X\left(f\right)e^{\left(2\pi ift\right)}df,$$

where X(f) represents the Fourier transform of the original input signal x(t), H(f) corresponds to the frequency response of the filter, and the integration is carried out over the specified frequency range of interest. Figure 2 illustrates the frequency response of the fourth-order bandpass filter designed and employed in this study.

Subsequently, the filtered signal y(t) undergoes a process of data normalization [46]. This normalization is employed to rectify any amplitude variations caused by factors such as changes in distance from the source or other influencing variables. Typically, normalization is executed by dividing the signal by its maximum absolute value, producing a normalized signal [46],

(2) $$z\left(t\right)=\frac{y(t)}{\max(|y(t)|)}.$$

Extracting meaningful information and characteristics from the collection of whale vocalization recordings is essential. Past works have demonstrated the effectiveness of conventional feature extraction and selection techniques in identifying and isolating key information from acoustic signals. These techniques have played a crucial role in analyzing underwater soundscapes, enabling researchers to extract meaningful patterns and characteristics essential for various applications. A comprehensive review of these methodologies highlights their significance in detecting and classifying whale species using machine learning algorithms.

### 2.4. Exisiting Feature Extraction Techniques for Whale Vocalizations

#### 2.4.1. PCA-Based Feature Extraction Method

The PCA is a generally utilized method for feature extraction and dimensionality reduction [19,20]. It is especially helpful for large and intricate datasets since it enables data simplification while preserving the most important information. PCA operates by determining the essential variables that significantly contribute to the variance inherent in the dataset [19]. This process is achieved through a transformation of the exisiting variables into new principal components (PCs). Each of these PCs comprises a linear combination of the initial variables, representing a distict type of variation in the dataset.

The PCs were arranged in order of importance such that the first reflected the most prominent contributor to variance within the data, while every other successive PC depicted the residual variance in a decreasing order of significance. The dataset’s dimensionality could be decreased while preserving the crucial information underlying the dataset by selecting only a subset of these PCs that collectively represented the majority of the data variability. This reduced the dimensionality of the dataset while retaining most of the information and was adopted with the HMM for whale detection in [20].

#### 2.4.2. DMD-Based Feature Extraction Method

DMD is a technique used to examine the temporal dynamics of complex systems, relying on empirical measurements to uncover underlying patterns. In 2010, Schmid introduced DMD [17] for analyzing complex flow fields, which involves isolating spatial modes and their corresponding temporal dynamics to reveal dominant patterns and forecast future behavior.

The first step of DMD is to represent the state of a system as a matrix such that snapshots in time are arranged in columns and spatial locations are represented by rows. The data matrix *X* is split into two matrices $X_{1}$ and $X_{2}$, where $X_{1}$ contains the first *m* columns of *X* and $X_{2}$ contains the remaining columns. Thereafter, the matrix $X_{1}$ is decomposed using singular value decomposition (SVD), yielding a low-rank approximation $U\Sigma V^{*}$. Here, *U* and *V* are orthogonal matrices, while Σ contains the singular values along its diagonal. In [22], the authors introduced kernel DMD for feature extraction from baleen whale vocalizations. Hidden Markov models were employed to evaluate the detection capabilities of the models. By modifying traditional DMD, the computational costs related to SVD mode extraction were decreased.

In [17], the DMD algorithm assumed that the system could be approximated by a linear operator acting on a set of spatial modes. This operator, called the Koopman operator, maps the state of the system at one time to the state of the system at a later time, represented by

(3) $$\begin{array}{c} A=X_{2}V\Sigma^{-1}U^{*}. \end{array}$$

Additionally, the DMD seeks to identify the dominant modes and corresponding frequencies of occurence, which are the eigenvectors ω and eigenvalues λ of the Koopman operator. The eigenvalue decomposition of *A* is computed as follows [17]:

(4) Aω=λω,

where ω is a nonzero vector and λ is a scalar. Therefore, the spatial modes or eigenvectors of *X* are computed by multiplying the eigenvectors ω by the columns of $X_{1}$ [17]:

(5) $$\begin{array}{c} \phi =X_{1}V\Sigma^{-1}\omega . \end{array}$$

Likewise, the temporal dynamics of each spatial mode is obtained by projecting the data onto the corresponding eigenmode as follows [17]:

(6) $$\begin{array}{c} b=\Phi^{+}x_{0}, \end{array}$$

where Φ is the matrix of spatial modes, $\Phi^{+}$ is the Moore–Penrose pseudoinverse of Φ, and $x_{0}$ is the initial state of the system. The resulting spatial modes and temporal dynamics can be used to analyze system behavior and make predictions about future behavior. The authors of [18,22] demonstrated using the extracted modes from the decomposition for whale data compression and dimensional reduction, thus adopting the modes with the HMMs to detect and classify whale vocalizations.

### 2.5. Wavelet Transform-Based Feature Extraction Method

The wavelet approach was introduced in [21], employing the wavelet transform (WT) as a feature extraction technique and effectively capturing the intricate time–frequency characteristics of a whale sounds. The WT decomposes the acoustic signals into various frequency components, allowing for a detailed examination of both temporal and spectral features. This decomposition is particularly advantageous for non-stationary signals like whale vocalizations, which exhibit dynamic frequency content over time.

The feature extraction process starts by computing the continuous wavelet transform (CWT) of a signal x(t) [47] by convolving x(t) with multiple scaled and time-shifted replicas of the mother wavelet ψ(t), which is as follows [47]:

(7) $$\mathrm{CWT}\left(a,b\right)=\frac{1}{\sqrt{\left|a\right|}}\int_{-\infty }^{\infty }x\left(t\right)\;\psi^{*}\left(\frac{t-b}{a}\right)dt.$$

Here, *a* and *b* denote the scale and translation parameters, respectively, and $\psi^{*}\left(t\right)$ represents the mother wavelet’s conjugate pair. Upon obtaining the CWT coefficients, three key features—the dispersion of energy across frequency bands, the centroid of the frequency spectrum, and the entropy of the wavelet coefficients—are extracted to encapsulate the salient characteristics of x(t).

The dispersion of energy across frequency bands is assessed by computing the squared modulus of wavelet decomposition coefficients ${\left|\mathrm{CWT}\left(a,b\right)\right|}^{2}$, highlighting the influence of individual spectral elements at varying resolutions and scales. To reduce noise and enhance interpretability, the energy values are smoothed using a moving average filter, given by [21]

(8) $$E\left(a,t\right)=\frac{1}{N}\sum_{i=-N/2}^{N/2}{\left|\mathrm{CWT}\left(a,t+i\right)\right|}^{2},$$

where *N* denotes the extent of the regularization interval. Subsequently, the magnitude values are rescaled utilizing robust range normalization, namely with the *z* score. This ensures uniformity and coherence across different scales and time points.

The centroid of the frequency spectrum helps determine the signal’s power concentration point during a given temporal interval, offering insights into the dominant spectral components. It is calculated as a weighted average of the frequencies, where the squared amplitude values of the wavelet transform coefficients dictate the relative influence of each frequency component [21]:

(9) $$F\left(t\right)=\frac{\sum_{a}f\left(a\right){\left|\mathrm{CWT}\left(a,t\right)\right|}^{2}}{\sum_{a}{\left|\mathrm{CWT}\left(a,t\right)\right|}^{2}}.$$

Here, f(a) denotes the spectral value associated with the scale parameter *a*. Variations in the frequency centroid over time can indicate changes in the prevailing spectral characteristics of the vocalizations.

The entropy of the wavelet coefficients characterizes the intricacy and unpredictability of the signal’s spectral-temporal representation. At every resolution level *a* and instant *t*, a statistical distribution is defined based on the normalized energy contributions [21]:

(10) $$p\left(a,t\right)=\frac{{\left|\mathrm{CWT}\left(a,t\right)\right|}^{2}}{\sum_{a^{\prime }}{\left|\mathrm{CWT}\left(a^{\prime },t\right)\right|}^{2}}.$$

Therefore, the entropy value at instant *t* is calculated as follows [21]:

(11) $$H\left(t\right)=-\sum_{a}p\left(a,t\right)\log_{2}p\left(a,t\right).$$

This measure reflects the distribution of energy across scales and provides insights into the signal’s complexity.

Finally, the derived feature—normalized energy distribution, centroid of the frequency spectrum, and entropy of the wavelet coefficients—are compiled into a feature matrix WF, which is structured as follows:

(12) $$WF=\left[\begin{array}{ccc} E_{1} & F_{1} & H_{1}\\ E_{2} & F_{2} & H_{2}\\ \vdots & \vdots & \vdots \\ E_{t} & F_{t} & H_{t} \end{array}\right].$$

In this matrix, each row corresponds to a specific time point *t*, and the columns represent the extracted features: the normalized energy distribution *E*, centroid of the frequency spectrum *F*, and entropy of the wavelet coefficients *H*. This comprehensive feature set serves as the input for subsequent classification stages, enabling the effective identification and analysis of a whale signal.

### 2.6. Proposed MSE-Based Feature Extraction Method

**Figure 3:**
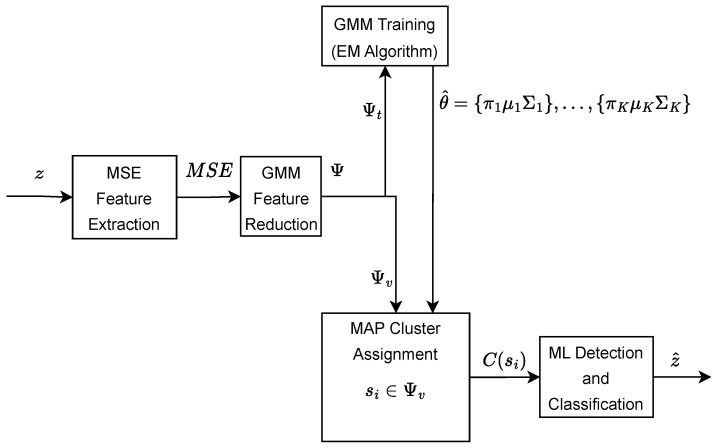
System model of the proposed MSE-GMM.

Figure 3 shows the framework of the proposed MSE-GMM detection and classification for blue whale vocalization.

This method extends the conventional SE algorithm by computing the sample entropy values at multiple scales, providing a more comprehensive analysis of the data. The MSE-GMM captures the dynamic behavior of the preprocessed signal at different time scales, making it useful for the analysis of non-stationary whale vocalizations. Each process of MSE computation and feature extraction is discussed below.

#### 2.6.1. Data Segmentation and Coarse-Grained Series Construction

The preprocessed blue whale dataset z(t) is segmented into *s*-dimensional, non-overlapping *t*-duration windows $F_{k}\in z\left(t\right),\;k=1,2,\ldots ,s$. This ensures that the complexity of the vocalization is analyzed over time and determines how the entropy measures respond to different stimuli or environmental conditions in the recordings. The length of each segment is determined by the parameters of the whale’s vocalization. For instance, the Z calls made by a blue whale vary in duration from 8 s to 12 s. Therefore, for a 3600-s annotated dataset, the segment length can be selected as a duration of 15 s. This implies that the signal would be segmented into s=240 non-overlapping 15-s segments.

A consecutive coarse-grained series is obtained by further dividing each segment into pairs of adjacent data points, depending on a scalar factor τ. For instance, if the segment is of a length L, then there will be τ frames of a length L/τ. Let $w_{i}$ denote the *i*th data point in a segment. Then, the coarse-grained time series for a given scale factor τ is constructed as follows [27]:

(13) $$c_{j}^{\left(\tau \right)}=\frac{1}{\tau }\sum_{i=(j-1)\tau +1}^{j\tau }w_{i},\;1\leq j\leq L/\tau .$$

This equation calculates the mean value of each window of a length τ and replaces the corresponding data points in the original segment $F_{k}$ with the mean value to obtain the coarse-grained series. If τ=1, then $c_{j}^{\left(\tau \right)}$ simply corresponds to the unaggregated data in the segment. Note that each temporal scale level represents the time series data at a coarser level of the vocalization signals and is used to extract information about the blue whale vocalization pattern at different scales. The coarse-graining procedure is repeated for all segments to obtain a series of coarse-grained time series for each scale and for each segment ${\left\{c_{j}^{\left(\tau \right)}\right\}}_{k}$.

#### 2.6.2. Feature Matrix Construction

Given ${\left\{c_{j}^{\left(\tau \right)}\right\}}_{k}$, the irregularity of the rescaled coarse-grained time series is measured at each scale based on the SE method [6,7,8]. At each coarse-grained scale $c_{j}^{\left(\eta \right)},\;\eta =1,2,\ldots ,\tau$, SE quantifies the likelihood that subseries of a length *m* which are similar within a tolerance level *r* remain similar when the length of the series is increased by one (i.e., m+1) [6]. The first step in SE-based feature extraction is to select the embedding dimension (*m*) and the threshold value (*r*). The embedding dimension determines the number of data points used to define a pattern or template [6]. It is a crucial parameter for calculating the sample entropy as a measure of the signal complexity, and thus *m* must be chosen with care [6,7,8]. In this study, the embedding dimension is chosen empirically such that it is sufficiently large to capture the dynamics of the blue whale signals but not too large to avoid overfitting.

The threshold value is the tolerance or maximum distance allowed between the patterns [6]. It is also an important parameter in SE-based feature extraction since it determines the similarity between two subseries. A small *r* value implies that two patterns are considered similar only if they are extremely close, while a larger *r* value allows more variability between patterns [6,7,8]. Similar to *m*, the value of *r* is usually selected empirically. Thus, in this study, it was chosen to be small enough to detect fine-grained patterns in the time series signal but not too small to avoid excessive noise. In the literature, the value of *r* is usually obtained as a fraction of the standard deviation σ of the data.

Embeddings are generated for the *k*th segment and each scale by creating a set of *m*-dimensional vectors, each of which consists of *m* consecutive values of the coarse-grained series $c_{j}^{\eta }$. Hence, an embedding space of a size M×m, where M=(L/τ)−m+1, is achieved by sliding a window of a size *m* along the coarse-grained series at time step *i* as follows:

(14) $$v_{i}=\left\{c_{j}^{\eta }\left(i\right),c_{j}^{\eta }\left(i+1\right),\ldots ,c_{j}^{\eta }\left(i+m-1\right)\right\}\;i=1,2,\ldots ,l.$$

Note that the window is slid one step at a time, and thus the next vector is obtained by shifting the window one step to the right.

The similarity between all pairs of the embedding vectors is computed without self-matching using the Chebyshev distance metric [6,48]. Let $E_{i}$ and $E_{j}$ be two *m*-dimensional embedding vectors for a segment $F_{k}$ and scale τ. The Chebyshev distance $d_{C}\left(E_{i},E_{j}\right)$ between $E_{i}$ and $E_{j}$ is given by [48]

(15) $$d_{C}\left(E_{i},E_{j}\right)=\max_{n=1}^{m}\left|E_{i}\left(n\right)-E_{j}\left(n\right)\right|$$

where $E_{i}\left(n\right)$ is the *n*th element of $E_{i}$ and $E_{j}\left(n\right)$ is the *n*th element of $E_{j}$. To determine the similarity between $E_{i}$ and $E_{j}$, $d_{C}\left(E_{i},E_{j}\right)$ is compared to the threshold value *r*. If $d_{C}\left(E_{i},E_{j}\right)\leq r$, then the pair $\left(E_{i},E_{j}\right)$ is considered to be similar. Otherwise, they are considered dissimilar.

In this study, we define a similarity matrix $D_{i,j}^{m}$ of a size M×M to store the computed Chebyshev distance between all pairs of *m*-dimensional vectors as follows:

(16) $$D_{i,j}^{m}=\left\{\begin{array}{cc} 1 & \mathrm{if}\;i\neq j\;\mathrm{and}\;d_{C}\left(E_{i},E_{j}\right)\leq r\\ 0, & \mathrm{otherwise}\; \end{array}\right..$$

The diagonal elements of *D* are set to zero, since the distance between a vector and itself does not have to be considered. Also, $D_{i,j}^{m}$ is a symmetric matrix, since the distance metric is symmetric. Therefore, it suffices to sum up the elements in the upper triangular part of the matrix $D_{i,j}^{m}$ while excluding the diagonal to compute the number of similar patterns $C^{m}$ within the tolerance *r*. In this way, the  number of pairs of m-dimensional embedding vectors within the tolerance *r* of each other can be counted. Thus, the expression for computing $C^{m}$ for each scale and segment is written as follows:

(17) $$C^{m}=\sum_{i=1}^{M-1}\sum_{j=i+1}^{M}\Theta \left(r-D^{m}\left(i,j\right)\right),$$

where Θ is the Heaviside step function that amounts to one if the argument is true and zero otherwise. This is equivalent to [27,28,49]

(18) $$C^{m}=\sum_{i=1}^{M-1}\sum_{j=i+1}^{M}\left[D^{m}\left(i,j\right)\leq r\right].$$

Note that the outer sum is summed up to M−1 to ensure that pairs which involve the last *m* embedding vectors are not counted, which would have less than *m* neighbors.

In addition to computing $C^{m}$, the number of m+1-dimensional vectors that match an *m*-dimensional vector within the tolerance *r* is also computed for each scale and segment, denoted by $B^{m+1}$. The purpose is to normalize $C^{m}$. To compute $B^{m+1}$, a new set of m+1-dimensional vectors is created by sliding a window of a length m+1 over the original coarse-grained series.

Similar to Equation (15), the Chebyshev distance is computed between all pairs of the m+1-dimensional embedding vectors and the *m*-dimensional embedding vectors for the same scale and segment. This results in a similarity matrix $D^{m+1}\left(ij\right)$ with dimensions M×(M+1). If the distance between an m+1-dimensional vector and an *m*-dimensional vector is less than or equal to *r*, then it is counted as a match. Hence, the expression for computing $B^{m+1}$ for each scale and segment is given as follows [49]:

(19) $$B^{m+1}=\sum_{i=1}^{M^{\prime \prime }}\sum_{j=i+1}^{M}1_{\left\{D^{m+1}\left(i,j\right)\leq r\right\}},$$

where $M^{\prime \prime }$ is the number of (m+1)-length vectors that can be formed from the original coarse-grained series, excluding the last element, and 1· is the indicator function that amounts to one if the argument is true and zero otherwise.

After computing the number of similar patterns $C^{m}$ and the number of matching m+1-dimensional vectors $B^{m+1}$, the probabilistic parameter $P^{m}$ is computed as follows [27,28,49]:

(20) $$P^{m}=\frac{C^{m}}{T^{m}},$$

where $T^{m}$ is the total number of *m*-dimensional vectors without self-matching, represented by [49]

(21) $$T^{m}=\frac{m(L/\tau -m+1)}{2}.$$

Similarly, the probabilistic parameter $P^{m+1}$ can be computed as follows [27,28,49]:

(22) $$P^{m+1}=\frac{C^{m+1}}{T^{m+1}},$$

where $T^{m+1}$ is the total number of m+1-dimensional vectors without self-matching, given by [49]

(23) $$T^{m+1}=\frac{\left(m+1)(L/\tau -m\right)}{2}.$$

These probabilistic parameters are therefore used to compute SE for each scale η=1,2,…,τ at the *k*th segment, given by [6]:

(24) $$SE_{k}^{\left(\eta \right)}=-\ln\left(\frac{P^{m+1}}{P^{m}}\right).$$

This represents the ratio of the probabilities of finding similar patterns in two vectors of the data segment that have lengths *m* and m+1. The lower the probability of finding similar patterns, the higher the sample entropy, indicating greater irregularity or complexity in the data.

Hence, the MSE matrix of the whale vocalizations F is obtained from the SE at each segment *k* as follows:

(25) $$MSE=\left[\begin{array}{cccc} SE_{11} & SE_{12} & \ldots & SE_{1\tau }\\ SE_{21} & SE_{22} & \ldots & SE_{2\tau }\\ \vdots & \vdots & \vdots & \vdots \\ SE_{s1} & SE_{s2} & \ldots & SE_{s\tau } \end{array}\right].$$

### 2.7. GMM-Based Feature Reduction

The s×τ dimensional feature matrix in Equation (25) contains *s* number of observations and τ features, and it is prepared for fitting the GMM. Here, the logarithm of the MSE features is taken to ensure that the features have a Gaussian distribution. This is useful for clustering and classification tasks because it allows for better separation of the different clusters or classes. In addition, the logarithmic transformation helps reduce the influence of outliers and makes the distribution of the feature values more symmetric [50], which can improve the performance of the GMM.

As described in [51,52], the GMM is utilized to perform feature reduction of the log transform of the feature matrix $MSE_{log}$ by identifying the most descriptive combination of features that capture the intrinsic patterns and relationships of the acoustic data. This is accomplished by fitting the GMM to the $MSE_{log}$ matrix and using the posterior probabilities of each data point to select the features that are most informative for clustering similar patterns. The optimal number of mixture components in the GMM is determined using the Bayesian information criterion (BIC) [53], which is defined as follows:

(26) BIC=−2log(L)+klog(n),

where *L* is the probability of observing the data set, *k* is the quantity of free parameters defining the model architecture—that is, the means, covariances, and mixture weights—and *n* is the total sample size. These parameters describe the clusters that are formed based on the input features.

The posterior probabilities of each data point are computed using Bayes’ rule [54,55]. This indicates the likelihood of association between the data point and each of the Gaussian components comprising the GMM model, which are obtained as follows [55]:

(27) $${\mathrm{posterior}}_{i}\left(j\right)=P\left(j|x_{i}\right)=\frac{P\left(x_{i}|j\right)P\left(j\right)}{\sum_{k}P\left(x_{i}|k\right)P\left(k\right)}$$

where *j* denotes the *j*th Gaussian component in the GMM, P(j) is the prior probability of the *j*th component, $P(x_{i}|j)$ is the likelihood of the data point $x_{i}$ given the *j*th component, and $\sum_{k}\left(P\left(x_{i}|k\right)P\left(k\right)\right)$ is the marginal likelihood of observing the data point. The likelihood $P(x_{i}|j)$ is computed using the Gaussian probability density function (PDF) for the *j*th component as follows [55]:

(28) $$P\left(x_{i}|j\right)=\frac{1}{\sqrt{{\left(2\pi \right)}^{d}\det\left(\Sigma_{j}\right)}}\exp\left(-\frac{1}{2}{\left(x_{i}-\mu_{j}\right)}^{T}\Sigma_{j}^{-1}\left(x_{i}-\mu_{j}\right)\right),$$

where $\mu_{j}$ is the mean vector of the *j*th component, $\Sigma_{j}$ is the covariance matrix of the *j*th component, and *d* is the dimensionality of the data. Also, the marginal likelihood $\sum_{k}\left(P\left(x_{i}|k\right)P\left(k\right)\right)$ is the weighted sum of the likelihoods of the data point given all of the Gaussian components in the GMM, represented by [55]

(29) $$\sum_{k}P\left(x_{i}|k\right)P\left(k\right)=\sum_{j}P\left(x_{i}|j\right)P\left(j\right).$$

The ranking of features is established based on their discriminatory power among the GMM components. This is performed by first computing the mean posterior probability of each component *j* for each feature *f* as follows:

(30) $$MPP\left(j,f\right)=\frac{1}{s}\sum_{i=1}^{s}{\mathrm{posterior}}_{i}\left(j\right)\cdot {\mathrm{feature}}_{i}\left(f\right),$$

where *s* represents the total sample size, corresponding to the number of rows in the feature matrix, ${\mathrm{posterior}}_{i}\left(j\right)$ denotes the posterior probability that data point *i* originates from component *j*, and ${\mathrm{feature}}_{i}\left(f\right)$ signifies the measured or computed value of a feature *f* associated with the *i*th data point. Thereafter, MPP is averaged across all features to obtain a single score that reflects the discriminative power of that component as follows [54]:

(31) $$score\left(j\right)=\frac{1}{\tau }\sum_{f=1}^{\tau }MPP\left(j,f\right),$$

where *j* is the GMM component index and τ is the number of features. The process is repeated for all GMM components, resulting in a ranking of the features based on their ability to discriminate between the different components. The features with the highest mean posterior probability for a given component are considered more informative for clustering and classification tasks. Hence, the top-ranked features are selected according to the desired number of features. A new feature matrix is created by retaining only the ζ columns corresponding to the selected features, which are given by

(32) $$\Psi =\left[\begin{array}{cccc} \nu_{11} & \nu_{12} & \ldots & \nu_{1\zeta }\\ \nu_{21} & \nu_{22} & \ldots & \nu_{2\zeta }\\ \vdots & \vdots & \vdots & \vdots \\ \nu_{s1} & \nu_{s2} & \ldots & \nu_{s\zeta } \end{array}\right].$$

### 2.8. GMM Training

The reduced feature matrix of the traning dataset $\Psi_{t}$ is used to fit a new GMM using similar mixture components with the original GMM. Let the new GMM’s parameters be denoted by θ with the following components [56]:

(33) $$\theta =\left\{\pi_{1},\mu_{1},\Sigma_{1},\pi_{2},\mu_{2},\Sigma_{2},\ldots ,\pi_{K},\mu_{K},\Sigma_{K}\right\},$$

where $\pi_{j}$ represents the coefficient of mixing the *j*th component, $\mu_{j}$ denotes the mean vector for the *j*th component, and $\Sigma_{j}$ is the matrix of covariance for a component *j*. The likelihood function for the new GMM can be expressed as follows [54,56]:

$$L\left(\theta |\Psi_{t}\right)=\prod_{i=1}^{s}\sum_{j=1}^{K}\pi_{j}N\left(\nu_{i}|\mu_{j},\Sigma_{j}\right).$$

In this equation, *K* represents the total number of GMM components, while $N(x_{i}|\mu_{j},\Sigma_{j})$ denotes the Gaussian probability density function evaluated for the $\nu_{i}$ data point, characterized by a mean vector $\mu_{j}$ and the covariance matrix $\Sigma_{j}$.

The EM algorithm [56] performs the maximum likelihood estimates of the parameters θ in an iterative process. The algorithm iteratively updates the values of θ until convergence is reached. The E step computes the posterior probabilities of each data point belonging to each component, while the M step updates the parameters of each component based on the posterior probabilities. The process is repeated until convergence is reached. Once the algorithm converges, the final updated GMM parameters are obtained as $\hat{\theta }$ and can be used for data testing and clustering new data points in the testing datasets.

### 2.9. GMM Testing, Clustering, and Classification

#### GMM Clustering

The estimated GMM parameters $\hat{\theta }$ are employed to represent the GMM’s fit to the reduced feature matrix of the testing data $\Psi_{v}$. The cluster assignment for each data point $s_{i}\in \Psi_{v}$ is performed based on the maximum a posteriori (MAP) classification rule [57], given by

(34) $$C\left(s_{i}\right)=\arg\max_{j}P\left(j|s_{i}\right),$$

where $P(j|s_{i})$ is the posterior probabilities for each data point in the testing set. The clustering assigns a new observation to the cluster with the highest posterior probability given the observation. This is repeated for all data points to obtain the final clustering of the data into *K* clusters.

### 2.10. GMM Classification

After the data points have been clustered, the GMM is used to perform classification by assigning a class label to a new data point based on its proximity to the cluster centers. This is performed using the maximum likelihood (ML) classification rule [58] based on the updated GMM parameters, where the new data point is assigned to the cluster with the highest likelihood. The predicted class label for the new data point *s* is expressed as follows:

(35) $$C_{ML}\left(s\right)=\arg\max_{j}p\left(s|\hat{\theta_{j}}\right),$$

where $p(s|\hat{\theta_{j}})$ is the likelihood of the new data point under the *j*th cluster.

Algorithm 1 gives a summary of the proposed GMM-based feature reduction and classification technique.
**Algorithm 1** GMM-based feature reduction and classification**Require:** MSE feature matrix MSE with *s* samples and τ features, number of GMM components *K*, number of top-ranked features ζ
1:Logarithmic transformation of the MSE feature matrix: $MSE_{log}=\log\left(1+MSE\right)$2:Fit a GMM with *K* components to $MSE_{log}$ using the posterior probabilities3:Rank the features based on their ability to discriminate between the GMM components by computing the mean posterior probability of each component for each feature as MPP(j,f)4:Obtain a single score that reflects the discriminative power of the component as score(j)5:Select the top ζ features based on a fixed number to obtain the reduced feature matrix Ψ6:Refit the GMM to samples of the training set $\Psi_{t}$, obtaining parameters $\theta =\pi_{1},\mu_{1},\Sigma_{1},\ldots ,\pi_{K},\mu_{K}\Sigma_{K}$ and posterior probabilities $P(j|s_{i})$ for each data point *i* and component *j*7:Compute the GMM parameter’s maximum likelihood estimates based on the EM method to obtain $\hat{\theta }$8:Cluster the data points into *K* distinct clusters according to the updated GMM parameter $\hat{\theta }$ using the MAP classification rule9:Classify samples of the testing set $\Psi_{v}$ using the ML classification rule based on the updated GMM parameters


## 3. Experimental Set-Ups

The proposed MSE-GMM’s performance is evaluated on large subsets of the dataset outlined in [30], specifically 8 h and above. The dataset library presents a vast and diverse collection of annotated audio recordings, totaling 1880.25 h of labeled data. This considerable dataset is the result of an extensive data collection effort, covering 11 site-years of recordings. Each site-year represents a unique combination of a single recording device and a specific survey location, with data collection spanning approximately 12 months. The dataset encompasses recordings from seven geographically distinct sites, resulting in a total of 105,161 annotations. However, it is important to note that the distribution of these annotations across the different sites is not uniform, indicating variations in data collection intensity and annotation efforts between sites.

For the purposes of this study, the Balleny Islands 2015 location was selected after taking into account the distribution and quantity of annotations at each site. This site has 923 BmAnt-A distributed over 62 days, 44 BmAnt-B across 12 days, 31 BmAnt-Z across 6 days, and 47 Bm-D across 7 days of manual annotations. This suggests that 30.2%, 5.9%, 2.9%, and 3.4% of the hours had BmAnt-A, BmAnt-B, BmAnt-Z, and Bm-D annotations, respectively. Each annotation consists of a 3600-s recording of blue whale vocalizations in wav format sampled at a 1-kHz sampling rate. The annotations within the library serve as the foundation for feature extraction, facilitating the identification of distinct whale call types, as well as training, testing, and evaluating the performance of detection models. Specifically, 70% of the dataset was allocated for training purposes, while the remaining 30% was reserved for the prupose of testing.

**Figure 4:**
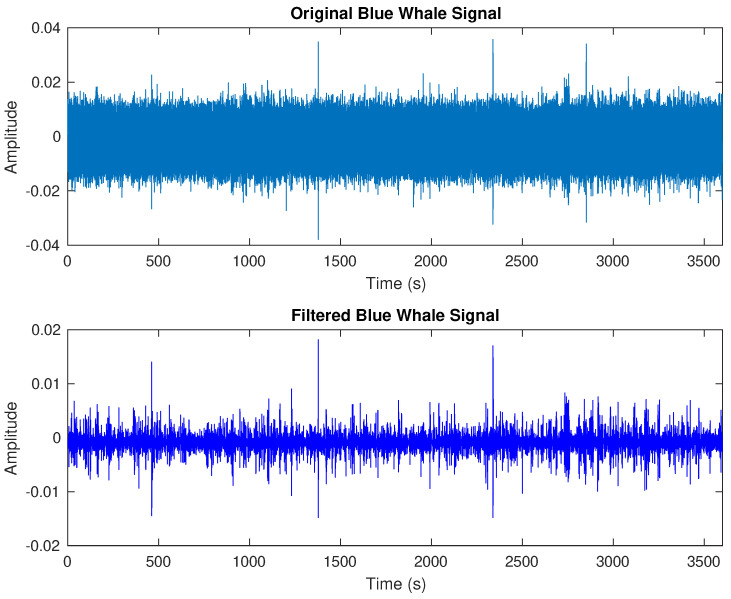
A comparison between the original and filtered blue whale signals.

The initial experimental process began by filtering the noise from the raw blue whale sound recordings. In Figure 4, a one-hour excerpt is presented, displaying both the original and filtered signals of blue whale vocalizations, as elaborated in [30,43].

### 3.1. Simulation Parameter Selection

It is important to select the appropriate parameters *m*, *r*, and τ to enhance the accuracy and reliability of the proposed MSE-based feature extraction method. In the literature, the value of *m* is generally chosen to be 2 [27], while *r* is selected as a factor of the standard deviation σ of the data, ranging between 0.2 and 0.5 [6]. In this study, the value of m=2 was empirically selected.

**Figure 5:**
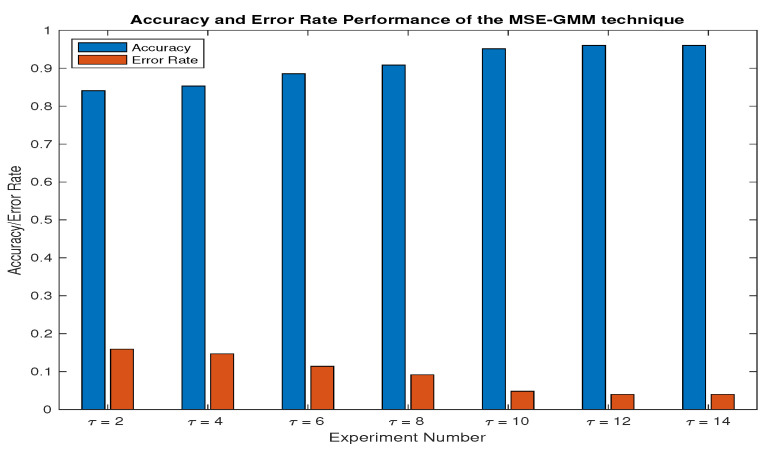
Antartic recording: maximum scaling value selection.

Given the characteristics of blue whale vocalizations, with call durations ranging between 8 and 12 s for BmAnt-A, 10 and 15 s for BMAnt-B, and 18 and 26 s for BmAnt-Z call types, a fixed window length of 26 s was selected for segmenting the acoustic recordings. To determine the optimal maximum scale factor τ, a range of values for τ was explored empirically. The acoustic recordings obtained from the Antartic sound recordings in [30] were analyzed using a range of τ values spanning from 2 and 14. The specific values for τ were selected based on their ability to maximize the accuracy and error rate performance of the proposed feature extraction technique, which leveraged MSE and the GMM as shown in Figure 5.

As depicted in Figure 5, the τ value of 12 displayed the most appropriate performance in contrast to the values of τ=2,4,6,8,and10. A larger τ value was required for the blue whale dataset due to the durations of the calls. The feature extraction capability of τ=12 was relatively comparable to that of τ=14. Thus, the maximum value of τ=12, yielding an accuracy performance of 96.02% and error rate performance of 3.98%, was chosen for the dataset.

### 3.2. Computational Complexity

A detailed comparison of the computational complexity of the PCA-GMM, DMD-GMM, WT-GMM, and proposed MSE-GMM methods is presented to highlight their computational efficiency and feasibility for large-scale datasets. Each method follows a distinct approach to feature extraction before applying the GMM for classification.

The PCA-GMM method primarily relies on computing the covariance matrix and performing eigendecomposition for dimensionality reduction. The computation of the covariance matrix for a dataset with *s* samples and *d* features requires $O(sd^{2})$, while the eigendecomposition introduces an additional complexity of $O(d^{3})$. Once the principal components are obtained, GMM-based detection and classification follow. The EM algorithm for GMM parameter estimation contributes a complexity of $O(Ksd^{3})$, where *K* is the number of Gaussian components. Similarly, posterior probability computation and feature selection introduce another $O\left(Ksd^{3}\right)+O\left(s\tau K\right)$, where τ is the number of features before reduction. Consequently, the total computational complexity of the PCA-GMM method is given by

$$O\left(sd^{2}\right)+O\left(d^{3}\right)+O\left(Ksd^{3}\right)+O\left(Ksd^{3}\right)+O\left(s\tau K\right).$$

The DMD-GMM approach follows a different paradigm, where feature extraction is based on modal decomposition techniques. The computational cost primarily arises from SVD and solving an eigenvalue problem. The SVD step has a complexity of $O(sd^{2})$, while the eigenvalue decomposition requires $O(d^{3})$. Similar to the PCA-GMM method, the extracted features are then processed using the GMM for feature selection, leading to an additional complexity of $O\left(Ksd^{3}\right)+O\left(Ksd^{3}\right)+O\left(s\tau K\right)$. As a result, the total computational complexity of the DMD-GMM approach is

$$O\left(sd^{2}\right)+O\left(d^{3}\right)+O\left(Ksd^{3}\right)+O\left(Ksd^{3}\right)+O\left(s\tau K\right),$$

making it computationally comparable to the PCA-GMM method.

In contrast, the WT-GMM method leverages a wavelet transform for feature extraction, which operates differently from matrix decomposition-based methods. The wavelet decomposition of a signal with a length *L* has a complexity of O(LlogL). When applied to *s* time-segmented windows, the total complexity of the wavelet transformation becomes O(sLlogL). Following this step, the extracted features undergo GMM parameter estimation, posterior probability computation, and feature selection, with each contributing $O(Ksd^{3})$, $O(Ksd^{3})$, and O(sτK), respectively. This results in an overall WT-GMM computational complexity given by

$$O\left(sL\log L\right)+O\left(Ksd^{3}\right)+O\left(Ksd^{3}\right)+O\left(s\tau K\right).$$

Compared with the PCA-GMM and DMD-GMM approaches, the WT-GMM method demonstrates lower computational overhead during feature extraction, making it more efficient for large-scale datasets.

The proposed MSE-GMM method introduces a multiscale entropy-based approach, which involves data segmentation, entropy computation, and GMM-based feature reduction. The first step, data segmentation and coarse-graining, has a complexity of O(L) per segment. The MSE computation is the most computationally demanding phase, consisting of three sub-processes: embedding vector construction with O(mL/τ), pairwise similarity computation with $O(m{\left(L/\tau \right)}^{2})$, and entropy calculation per segment with O(L/τ). As a result, the total MSE complexity for the entire dataset is

$$O(s{\left(L/\tau \right)}^{2}m).$$

Similar to the other methods, the extracted features are processed using the GMM, where parameter estimation and posterior probability computation each contribute $O(Ksd^{3})$, while feature selection has a complexity of O(sτK). This leads to the total computational complexity for the MSE-GMM method being expressed as follows:

$$O\left(s{\left(L/\tau \right)}^{2}m\right)+O\left(Ksd^{3}\right)+O\left(Ksd^{3}\right)+O\left(s\tau K\right).$$

When comparing these methods, the PCA-GMM and DMD-GMM methods exhibit similar computational complexities due to their reliance on matrix decomposition techniques, with both requiring $O\left(sd^{2}\right)+O\left(d^{3}\right)$ for feature extraction. The WT-GMM approach, on the other hand, demonstrates lower complexity in feature extraction with O(sLlogL), making it computationally efficient for large datasets. However, the MSE-GMM approach incurs a significantly higher computational cost due to the pairwise similarity estimation in sample entropy, which scales as $O(s{\left(L/\tau \right)}^{2}m)$. The dominant computational terms in the MSE-GMM method arise from sample entropy computation and GMM parameter estimation, making it computationally more intensive than the PCA-GMM, DMD-GMM, and WT-GMM approaches. Despite its higher complexity, the MSE-GMM method provides a more detailed analysis of the data’s structure and variability, making it a powerful tool for feature extraction in applications that require robust signal characterization.

The following tables provide a summary of the computational complexity analysis of the different feature extraction methods integrated with the GMM. Table 1 presents a step-by-step breakdown of the computational cost associated with each processing step in all of the methods, while Table 2 provides an overall complexity comparison.

## 4. Results and Discussion

### 4.1. Correlation Analysis Between Features and Target Class

**Figure 6:**
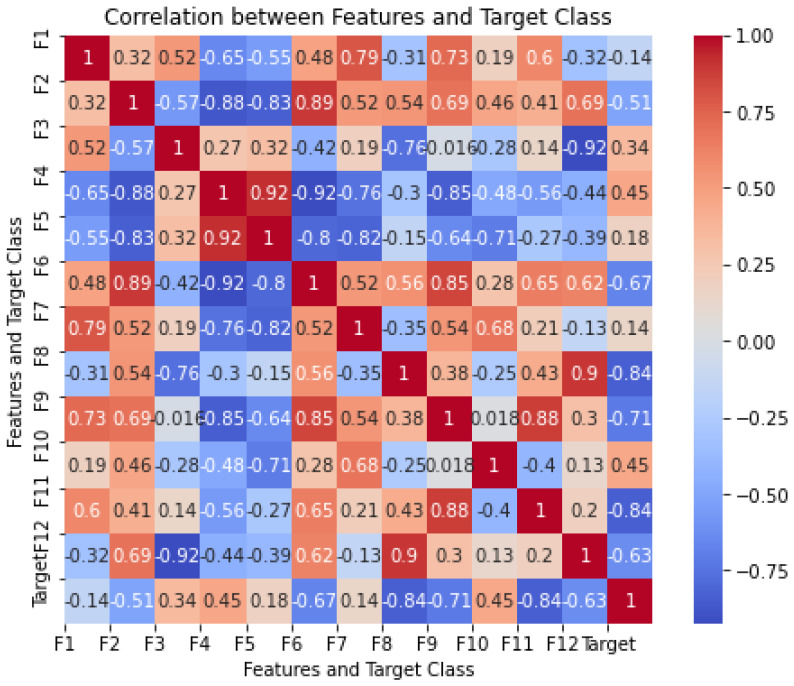
Correlation heatmap for blue whale vocalization dataset.

Figure 6 shows the correlation analysis between the τ features (τ=12) and the target class of the blue whale’s vocalizations. This provides significant insights into the relationships within the dataset. The correlation coefficients, ranging from −1 to 1, indicate the strength and direction of the linear relationship between each feature and the target class, as well as the inter-feature correlations.

Some features demonstrated strong correlations with the target class, indicating their potential predictive relevance. Notably, feature 4 (F4) and feature 10 (F10) exhibited the highest positive correlation, suggesting a strong linear relationship and their significance in predicting the target class, making them valuable predictors for the model. Similarly, feature 3 (F3) showed relatively high positive correlations, further suggesting its significance in predicting the target class.

Conversely, other features displayed weak or negative correlations with the target. Features 8 (F8) and 11 (F11) indicated the strongest negative correlation, implying an inverse relationship with the target class. Moreover, feature 2 (F2), feature 6 (F6), feature 9 (F9), and feature 12 (F12) had a relatively strong negative correlation, further showing an inverse relationship with the target class. Features like F1, F5, and F7 demonstrated weaker correlation, suggesting limited individual predictive power. However, despite their weak correlations, these features might still capture nonlinear or interaction effects, which could be beneficial when used alongside other variables.

The correlation matrix also highlights instances of strong inter-feature correlations. For example, F4 and F5 exhibited a high positive correlation, indicating possible redundancy. Such multicollinearity can inflate variance and reduce model interpretability. Additionally, F10 and F7 showed a strong correlation, suggesting that these features may provide overlapping information. Hence, feature reduction methods such as the PCA, DMD, and the GMM-based feature reduction methods are applied to mitigate redundancy. Addressing multicollinearity is essential to ensure model interpretability and minimize inflated variance.

On the other hand, some features demonstrated lower inter-feature correlations, indicating that they provide distinct information. Features with moderate correlations, such as F3 and F9, may add value without introducing redundancy. This balance is essential for the GMM’s performance, as retaining only unique and informative features is crucial for achieving generalizability.

### 4.2. Detection and Classification Performance Comparison

This study undertook an evaluation of the developed MSE-GMM method, assessing its performance in extracting discriminative features and detecting and classifying blue whale Z calls. The performance of the MSE-GMM approach was compared against two existing feature extraction techniques, PCA and DMD, which were adopted with the GMM. A comparative analysis was conducted, comparing the MSE-GMM technique to two prominent feature extraction methodologies: PCA and DMD. Notably, both PCA and DMD were integrated with the GMM, enabling a fair and informed comparison of the three approaches.

The ability of the schemes to detect whale vocalizations is demonstrated based on the accuracy and error rate performance metrics. Accuracy measures how often the algorithm correctly identifies a blue whale call, while the error rate indicates the percentage of times the algorithm fails to identify the call. For evaluating the prediction’s accuracy, the following metrics were utilized: true positives (TP), true negatives (TN), false positives (FP), and false negatives (FN). These quantified the number of accurate and inaccurate predictions. Accuracy was computed as follows [13]:

(36) $$\begin{array}{c} Accuracy=\frac{TP+TN}{TP+FP+TN+FN}, \end{array}$$

while the error rate is given by [13]

(37) $$\begin{array}{c} E=\frac{FN+FP}{TP+FP+TN+FN}. \end{array}$$

### 4.3. Detection Performance Comparison

Table 3 provides a comparative analysis of the performance results for the blue whale vocalizations, highlighting the key findings of the evaluation. The feature extraction, detection, and classification accuracy and error rate performance of the MSE-GMM, PCA-GMM, and DMD-GMM methods were quantified by their average classification rates, which were calculated over multiple iterations of the experiment.

**Figure 7:**
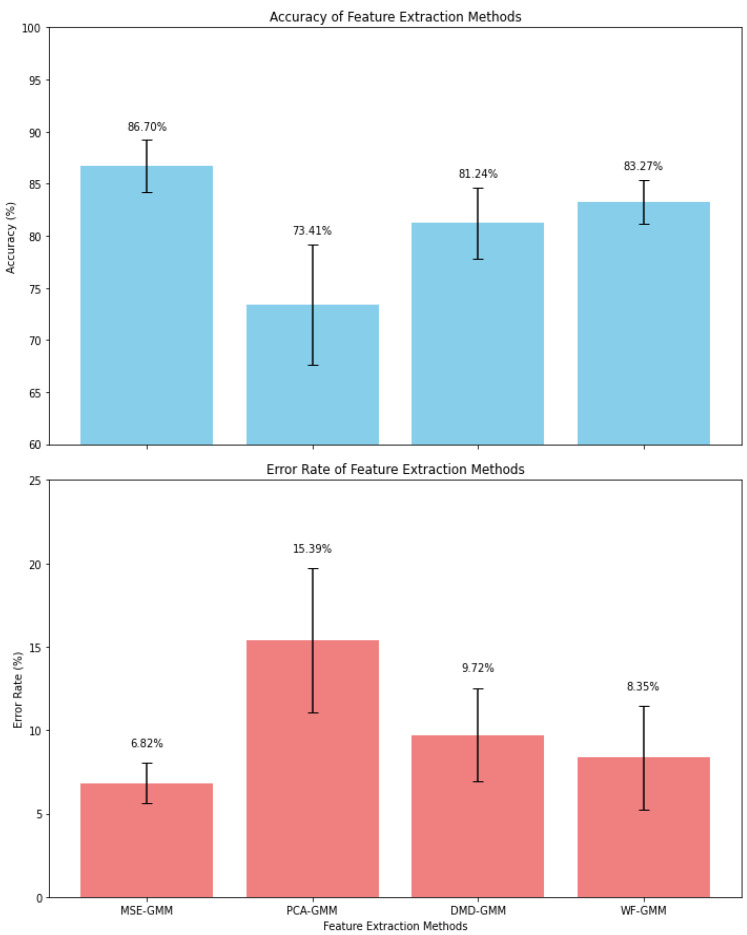
Performance comparison of feature extraction methods over 10 runs.

The results in Table 3 and Figure 7 reveal that the MSE-GMM algorithm outperformed the other methods, achieving an average accuracy of 86.20% with a peak of 88.15% and a low of 83.26%. Correspondingly, it recorded the lowest average error rate at 6.82%, fluctuating between 4.12% and 8.50%. Notably, the feature dimensionality for the MSE-GMM method was ζ=4.

The DMD-GMM approach demonstrated moderate performance, securing an average accuracy of 81.24%, with a high of 86.47% and a low of 77.96%. Its average error rate was 9.72%, varying between 5.11% and 14.25%. The feature dimensionality here was ζ=5.

Similarly, the WF-GMM method achieved an average accuracy of 83.77%, peaking at 85.01% and bottoming out at 81.95%. The corresponding average error rate was 8.35%, with a span from 4.53% to 12.69%. Notably, the WF-GMM approach had the lowest feature dimensionality among the methods, with ζ=3.

In contrast, the PCA-GMM method exhibited the least favorable performance with an average accuracy of 73.41%, reaching a maximum of 81.01% and dipping to a minimum of 69.42%. Its average error rate stood at 15.39%, ranging from 9.89% to 21.63%. The feature dimensionality for the PCA-GMM method was higher at ζ=7.

### 4.4. Discussion

The superior performance of the MSE-GMM algorithm can be attributed to its proficiency in capturing the intricate, non-stationary characteristics of blue whale vocalizations. The MSE-based feature extraction method effectively handled time-dependent data structures and encapsulated the nonlinear relationships inherent in the vocalization patterns.

In contrast, the PCA-GMM approach exhibited the least favorable performance. This outcome was due to PCA’s underlying assumptions of stationarity and homogeneity of variances, which are violated by the complex and dynamic nature of blue whale vocalizations. Consequently, PCA’s capacity to capture the data’s complexity and nonlinearity was diminished, leading to suboptimal detection and classification results.

The DMD-GMM method, while outperforming the PCA-GMM method, did not achieve the accuracy levels of the MSE-GMM approach. DMD is adept at analyzing dynamic systems and can capture temporal patterns. However, it may not fully encapsulate the nonlinear aspects of the vocalizations as effectively as MSE-based methods.

The WF-GMM approach demonstrated commendable performance, with an average accuracy of 83.77% and an error rate of 8.35%. Its lower feature dimensionality (ζ=3) suggests a more efficient model with reduced computational complexity, making it a viable alternative for real-time applications.

Considering computational complexity, the feature dimensionality (ζ) plays a crucial role. The MSE-GMM algorithm, with ζ=4, offered a balance between performance and computational efficiency. In contrast, PCA-GMM’s higher dimensionality (ζ=7) contributed to increased computational demands without corresponding performance benefits.

These findings underscore the importance of selecting appropriate feature extraction methods tailored to the specific characteristics of the data. The MSE-GMM algorithm’s ability to manage non-stationary and complex vocalization patterns, coupled with the relatively low feature dimensionality, makes it well suited for real-time applications. The WF-GMM approach also presents a promising alternative, offering a good balance between accuracy and computational efficiency. Future work could explore hybrid models that combine the strengths of these methods to further enhance detection and classification performance.

## 5. Conclusions

In this study, the MSE algorithm was presented as a time-domain feature extraction method for the purpose of investigating blue whale vocalization patterns in extended acoustic datasets. To enhance detection and classification capabilities, the MSE was integrated with the GMM framework, which was specifically tailored for identifying blue whale *Z* calls. To validate the effectiveness of the proposed MSE-GMM method, its performance was experimentally evaluated and benchmarked against three state-of-the-art methods—PCA, DMD, and WF—all of which were integrated with the GMM. Antartic sound recording open-source data were utilized to assess the performance of the MSE-GMM, PCA-GMM, DMD-GMM, and WF-GMM approaches for feature selection, detection, and classification of blue whale vocalization. The results showed that the proposed method outperformed the conventional PCA-GMM, DMD-GMM, and WF-GMM methods in terms of accuracy and error rate when detecting and classifying the non-stationary and complex vocalizations of blue whales. Notably, the MSE-GMM method also utilized a minimal feature dimension, making it compatible with real-time application requirements with low computational cost. A possible area for future work is to investigate the applicability and effectiveness of the proposed MSE-GMM approach with other acoustic datasets beyond the blue whale vocalization dataset used in this study. This could provide valuable insights into the generalizability and robustness of the method.

## Figures and Tables

**Table 1:** Comparative computational complexity analysis of PCA-GMM, DMD-GMM, WT-GMM, and MSE-GMM.

Processing Step	PCA-GMM	DMD-GMM	WT-GMM	MSE-GMM
Data segmentation	N/A	N/A	N/A	O(L) per segment
Feature transformation	$O(sd^{2})$	$O(sd^{2})$	O(sLlogL)	$O\left(m\frac{L}{\tau }\right)$
Feature extraction	$O(d^{3})$	$O(d^{3})$	O(sLlogL)	$O\left(m{\left(\frac{L}{\tau }\right)}^{2}\right)$
Feature selection	$O(d^{3})$	$O(d^{3})$	O(sLlogL)	$O\left(\frac{L}{\tau }\right)$
Total feature extraction complexity	$O\left(sd^{2}\right)+O\left(d^{3}\right)$	$O\left(sd^{2}\right)+O\left(d^{3}\right)$	O(sLlogL)	$O\left(s{\left(\frac{L}{\tau }\right)}^{2}m\right)$
GMM parameter estimation (EM)	$O(Ksd^{3})$	$O(Ksd^{3})$	$O(Ksd^{3})$	$O(Ksd^{3})$
Posterior probability computation	$O(Ksd^{3})$	$O(Ksd^{3})$	$O(Ksd^{3})$	$O(Ksd^{3})$
Feature selection via MPP ranking	O(sτK)	O(sτK)	O(sτK)	O(sτK)

**Table 2:** Overall computational complexity comparison of PCA-GMM, DMD-GMM, WT-GMM, and MSE-GMM.

Method	Computational Complexity
PCA-GMM	$O\left(sd^{2}\right)+O\left(d^{3}\right)+O\left(Ksd^{3}\right)+O\left(Ksd^{3}\right)+O\left(s\tau K\right)$
DMD-GMM	$O\left(sd^{2}\right)+O\left(d^{3}\right)+O\left(Ksd^{3}\right)+O\left(Ksd^{3}\right)+O\left(s\tau K\right)$
WT-GMM	$O\left(sL\log L\right)+O\left(Ksd^{3}\right)+O\left(Ksd^{3}\right)+O\left(s\tau K\right)$
MSE-GMM	$O\left(s{\left(L/\tau \right)}^{2}m\right)+O\left(Ksd^{3}\right)+O\left(Ksd^{3}\right)+O\left(s\tau K\right)$

**Table 3:** Performance comparison of MSE-GMM, PCA-GMM, and DMD-GMM (τ=12).

	Accuracy Across 10 Trials (%)			Error Rate Across 10 Trials (%)			ζ
	Mean	Highest	Lowest	Mean	Highest	Lowest	
MSE-GMM	86.20	88.15	83.26	6.82	8.50	4.12	4
PCA-GMM	73.41	81.01	69.42	15.39	21.63	9.89	7
DMD-GMM	81.24	86.47	77.96	9.72	14.25	5.11	5
WF-GMM	83.77	85.01	81.95	8.35	12.69	4.53	3

## Data Availability

This study analyzed publicly available datasets. The data are available at http://data.aad.gov.au/metadata/records/AcousticTrends_BlueFinLibrary (accessed on 1 February 2023).
